# A Trade as a Refuge

**Published:** 1885-03

**Authors:** 


### A TRADE AS A REFUGE.

Many years ago the writer was foreman of a machine shop in Boston, Mass., and one day had an application for apprenticeship from a young man who was accompanied by his uncle. The latter carefully explained that his nephew did not expect to be a machinist for a living, as there was ample means for his support outside of the workshop; but he wanted to learn the trade, so as to be independent of circumstances. The propriety of the intention of the young man and his uncle was recognized; but the exaction was made that the apprentice should travel the same road that impecunious and needy young men traveled; there was no royal road or short cut to mechanical success for lads of great expectations. These plain truths—very plainly presented—suited the applicant and his relative, who was at that time a United States Senator, and subsequently became a candidate for a still higher office.

The young man came into the shop, was treated the same as the other apprentices, was instructed as though he was to become a machinist and follow the honorable business for a living. But he disregarded shop hours; he sneered at shop rules: he came and went as he chose; and finally, six years after, he was usher at a second rate theatre. He was not cut out for an amateur mechanic.

His experience as an embryo mechanic illustrates the nonsense frequently talked in public and published in print—that the experimental knowledge of a trade or business is sure defense against possible disaster, and secures the journeyman apprentice a chance for an income from his trade. The notion is as fallacious as would be that of every graduate from a college claiming the qualifications for a professor.

It is well enough that young men should learn some means of supporting themselves by their own exertions, but it is folly to imagine that because a boy has soiled his overalls against a lathe and dirtied his hands with oil and filings, he is necessarily a mechanic and can return to his shop, as to a "city of refuge" when misfortune overtakes him.

No mechanic is worthy the name who does not keep abreast with the improvements in the shops. To do so, he must either work in the shop or be a frequent visitor. It is astonishing to men—practical mechanics—who write for publication to their brother mechanics, to see how the changes and possible improvements in shop methods and shop tools keep apace with their growing years. The sixty year old proprietor of a well known manufactory said, recently, that he was surprised every day when he compared what was being done in his own establishment with what he knew how to do thirty-five years ago; and this man is one of the liveliest mechanics and prolific inventors of the country. It is evident from observation, and it is convincing from experience, that a learned trade should be a practiced trade to be of real use.—*Scientific American.*

